# Cetuximab-modified silica nanoparticle loaded with ICG for tumor-targeted combinational therapy of breast cancer

**DOI:** 10.1080/10717544.2018.1564403

**Published:** 2019-02-23

**Authors:** Xiaoxue Zhang, Yinyan Li, Minjie Wei, Chang Liu, Jun Yang

**Affiliations:** a Department of Cardiovascular Ultrasonic Diagnosis, the First Affiliated Hospital of China Medical University, Shenyang, China;; b Department of Ultrasonic Diagnosis, the First Affiliated Hospital of China Medical University, Shenyang, China;; c Department of Pharmacology, School of Pharmacy, China Medical University, Shenyang, China;; d Department of Radiation Oncology, the First Affiliated Hospital of China Medical University, Shenyang, China

**Keywords:** Cetuximab, silica nanoparticles, indocyanine green, chemo-photothermal therapy, breast cancer

## Abstract

Combinational therapy is usually considered as a preferable approach for effective cancer therapy. Especially, combinational chemo and photothermal therapy is of particular interest due to its high flexibility as well as efficiency. In this article, we the silica nanoparticles (SLN) were surface conjugated with Cetuximab (Cet-SLN) to target epidermal growth factor receptor (EGFR), a common receptor that usually observed to overexpress in multiple breast cancers. Moreover, the high drug loading capacity of Cet-SLN was employed to encapsulate photothermal agent indocyanine green (ICG) to finally fabricate a versatile drug delivery system (DDS) able to co-deliver Cet and ICG (Cet-SLN/ICG) for combinational chemo-photothermal therapy of breast cancer. The obtained results clearly demonstrated that Cet-SLN/ICG was well-dispersed nanoparticles with preferable stability under physiological condition. Furthermore, due to the conjugation of Cet, Cet-SLN/ICG could target EGFR which overexpress in MCF-7 cells. Most importantly, both *in vitro* and *in vivo* results suggested that compared with Cet or ICG alone, the Cet-SLN/ICG showed superior anticancer efficacy. In conclusion, Cet-SLN/ICG could be a potential platform for effective combinational chemo-photothermal therapy for breast cancer.

## Introduction

To date, it has been generally recognized that nanoparticles based drug delivery systems (DDS) with enhanced bioavailability as well as reduced side effects hold promising anticancer effect as compared to free drugs (Wang et al., 2018). As a result, in the past decades, various nanoparticles based DDSs have been successfully developed using different materials including both organic and inorganic materials (Pérez-Ortiz et al., 2017; Wang et al., 2017; Zhao et al., 2018; Hongshuai et al., 2019). Silica nanoparticles (SLN) with versatile merits such as ease of fabrication, decent drug loading capacity as well as biocompatibility, is emerging as a preferable candidate (Daryasari et al., 2016; Martínezcarmona et al., 2016). Therefore, many DDSs using SLN as the skeleton have been developed and achieved satisfying outcomes both *in vitro* and *in vivo* (Wang et al., 2016; Duo et al., 2017; Li et al., 2017).

However, many currently available SLN derived DDSs still suffered from some inherent drawbacks, such as untargeted delivery as well as slow drug release that requires further improvements (You et al., 2017; Tang et al., 2018; Wang et al., 2018). To address the targeting dilemma, the most adopted approach is to modify DDSs with targeting ligands which can bind with corresponding receptors on the surface of cancer cell (Knezevic et al., 2016; Sun et al., 2017). Moreover, with the aim to accelerate the targeted drug release, the high glutathione (GSH) concentration within cancer cells was employed as stimulant. Previous articles have proved that DDSs with disulfide bonds can respond to GSH within cancer cells to achieve enhanced drug release (Du et al., 2015; Liu et al., 2015) DDSs combines both strategies have shown to have superior performance than those without (Zhao et al., 2014; Cheng et al., 2017).

Photothermal therapy (PTT) is a recently emerging approach that relies on photothermal agents to absorb NIR light, transfer it into heat and cause cytotoxic effects. It has been identified as a noninvasive and harmless technique with high efficiency in cancer therapy (Johnson and Pavelec, 1973; Hou et al., 2017). Indocyanine green (ICG) is known as the only PTT agent approved by the FDA for clinical imaging and diagnosis, which has many advantages over other competitors. However, limitations such as irreversible rapid degradation, short blood half-life as well as lack of targeting capacity strictly require the aid of additional DDSs for its further application in cancer therapy (Ding et al., 2017). Recently, monoclonal antibodies that can target corresponding receptors on the surface of cancer cells to exert specific functions are widely recognized as promising candidates for chemotherapy of cancer (Shuang et al., 2016; Colzani et al., 2018). Cetuximab (Cet) is a commonly adopted monoclonal antibody that targets epidermal growth factor receptor (EGFR) to inhibits the EGF signaling in cancer cells (Wang et al., 2017). The potential application of Cet in breast cancer therapy has been widely proposed and demonstrated to be positive (Brockhoff et al., 2007).

The monotherapy for cancer therapy is usually subjected to some insurmountable shortages, such as limited therapeutic benefits and strong systemic toxicity while combination therapy is considered as an alternative protocol to overcome this dilemma by simultaneously modulating different therapeutic pathways (Yu et al., 2017; Xie et al., 2018). As a result, the combination treatments of chemo and photothermal therapies to elevate the therapeutic benefits have attracted a great interest in scientific research (Huang et al., 2017). It has been generally recognized that maximal cooperation effect of combination therapy usually requires accurate doses of both agents to be simultaneously delivered to the same cancer cells using the same vector, which calls for the assistance of DDSs (Zheng et al., 2013).

In order to combine Cet and ICG for advanced chemo-PTT in one DDS with tumor targetability, thiolated SLN was synthesized and subsequently conjugated with Cet by disulfide bond to fabricate a tumor-targeted platform (Cet-SLN). The obtained Cet-SLN was finally loaded with ICG to obtain Cet-SLN/ICG. It was expected that Cet on the surface of the platform can specifically direct the Cet-SLN/ICG to the EGFR overexpressed MCF-7 cell line to increase its tumor-homing property and cellular uptake efficacy. After internalization by cancer cells, the disulfide bond could respond to the cytoplasmic GSH to trigger the fast release of Cet and ICG. Upon irritating with additional NIR light, both drugs could exert synergic effect to achieve preferable anticancer effects.

## Materials and methods

### Materials

(3-mercaptopropyl)-trimethoxysilane (MPTMS), β-mercaptoethylamine (MEA), Triton X-100 and tetraethyl orthosilicate (TEOS) were purchased from Sigma-Aldrich (St. Louis, MO) Cetuximab (Cet) was a gift from Merch KgaA (Frankfurter, Germany). Methyl thiazolyl tetrazolium (MTT) and glutathione (GSH) were obtained from Aladdin Co., Ltd (Shanghai, China).

### Cell culture and animal model

The MCF-7 cell line (human breast carcinoma) was obtained from American Type Culture Collection and cultured in standard condition as reported previously (Meng et al., 2018).

Female Balb/c nude mice (6–8 week) purchased from Model Animal Research Center of Nanjing University (Nanjing, China) were raised in SPF-II condition (25 ± 2 °C) with access to diet at liberty. To the establishment of MCF-7 tumor xenograft model was performed according to previous article (Li et al., 2018). Once the tumor volume reached the threshold of 100 mm^3^, mice were recruited for *in vivo* experiments. All animal procedures were approved by institutional Ethics Committee of the First Affiliated Hospital of China Medical University.

### Preparation of Cet-SLN/ICG

The preparation of thiolated SLN was performed in a water-in-oil microemulsion according to previous report (Wan et al., 2017) while thiolated Cet was synthesized strictly in accordance with previous literature (Wang et al., 2017).

To obtain Cet-SLN, 0.5 mL of thiolated Cet (aqueous solution, 2 mg/mL) was added drop-wise into 5 mL of thiolated SLN (aqueous solution, 2 mg/mL). The mixture was gently stirred at room temperature for another 8 h to allow reaction. The resulted Cet-SLN was obtained using centrifugation at 6000×*g* for 15 min (ALLEGRA X-15R, Beckman).

The conjugation efficacy of Cet in Cet-SLN was determined by calculating the remaining Cet in the supernatant using a Bradford kit (Thermo Fisher). All procedures followed the manufacturer’s instruction with a microplate reader (FlexStation 3, Molecular Devices).

Fluorescence investigation was performed as previously reported (Liao et al., 2011), to verify the successful conjugation of Cet. In brief, FITC-conjugated rabbit-anti-human IgG (Invitrogen) was incubated with of Cet-SLN/ICG with 0.9% bovine serum albumin at room temperature for 1 h. Afterward, the mixed solution was being centrifuged at 8000×*g* for 10 min. The obtained precipitate was re-dispersed in 20 μL of PBS and observed under fluorescence microscope (IX51, Olympus, Japan).

Afterward, the obtained Cet-SLN was re-suspended in water (2 mg/mL), followed by drop-wise adding of ICG (aqueous solution, 0.5 mL, 2 mg/mL) under gentle agitation. After 30 min of incubation, the mixture was sonicated using a probe-type ultrasonicator (VCX130PB, Sonics, 200 W, 15 min). The Cet-SLN/ICG was isolated by centrifugation (56000×*g*, 15 min). The drug loading content (DLC) of Cet-SLN/ICG was determined by calculating the ICG in the supernatant at 781 nm by UV spectrophotometer (V1100D, MAPADA, China) according to the following formula:

DLC (wt%) ＝ (weight of loaded ICG/weight of Cet−SLN/ICG)×100%

### Characterization

To confirm the successful modification of Cet onto SLN, fluorescence method was performed according to a previous report (Liao et al., 2011). The size distribution of Cet-SLN/ICG was assessed by a size analyzer (LS 13 320, Beckman Coulter). The zeta potential was assessed by a zeta analyzer (90PlusZeta, Brookhaven). Transmission electron microscope (TEM, JEM-2100, JEOL, Japan) was applied to observe the morphology of Cet-SLN/ICG.

### Stability assay

The freshly prepared Cet-SLN/ICG was diluted using phosphate buffer (PBS, pH 7.4, 6.8 and 5.5, 1:10, w/w). The colloidal stability of the system was observed by monitoring the change in particle size for up to 48 h. For fluorescence stability test, the fluorescence intensity of Cet-SLN/ICG was continuously monitored for 6 days under sunlight using fluorescence spectrophotometer (F-7000, Hitachi, Japan) with excitation at 740 nm and emission at 815 nm. The change in fluorescence intensity in comparison to free ICG was plotted (Ding et al., 2017).

### *In vitro* release and photothermal conversion experiments

The release profile of Cet and ICG from Cet-SLN/ICG, respectively, was investigated according to previous report (Meng et al., 2018).

The photothermal conversion assay was also studied. In brief, 2 mL of Cet-SLN/ICG solution was irradiated by 808 nm laser (1 W/cm^2^) with free ICG and PBS as controls. At the same time, the changes of temperature as a function of time was recorded by a digital thermometer.

### Cytotoxicity assay

The cytotoxicity effect of drug free nanoparticles (5–100 μg/mL) as well as Cet-SLN/ICG (ICG concentration, 0.25–5 μg/mL, with or without laser irritation, 2 W/cm^2^ for 5 min) on MCF-7 cells for 48 h was studied using a standard MTT assay as reported previously (Wang et al., 2017).

### Cellular uptake assay of Cet-SLN/ICG

MCF-7 cells in six-well plates with 70% confluence were cultured with different formulations for designated time intervals. Additionally, to explore whether Cet can increase the uptake efficacy of Cet-SLN/ICG, prior to sample addition, cells were incubated with excess Cet (100 μg/mL) for 2 h. At prearranged time intervals, cells were detached, harvested and then assessed by quantitative analysis of intracellular mean fluorescence intensity by flow cytometer (FCM, Attune NxT, Thermo Fisher).

### *In vivo* tumor targeting of Cet-SLN/ICG

Mice bearing MCF-7 tumor were intravenously injected with Cet-SLN/ICG and SLN/ICG (5 μg/mouse of ICG). At 48 h post administration, the mice were sacrificed to obtain tumor tissues as well as major organs. The *in vivo* biodistribution of the two formulations was evaluated using real-time imaging system (ZEWTON 7.0, Vilber, France).

### *In vivo* antitumor assay

*In vivo* antitumor assay of Cet-SLN/ICG was assessed using MCF-7 tumor-bearing mice. In detail, mice were randomly divided into four groups (*n* = 6): (1) saline (as control); (2) free ICG with laser irritation (1 W/cm^2^ for 5 min); (3) Cet-SLN; (4) Cet-SLN/ICG with laser irritation. Protocols were adopted from previous report (Colzani et al., 2018). Briefly, mice were administrated via tail vein (2 mg/kg ICG and/or 5 mg/kg Cet per mouse) for seven times in a 14-day period. The tumor volume and body weight were monitored once every 2 days. At the end of assay, the obtained tumor tissues from sacrificed mice were subjected to standard TUNEL staining and imaged under microscope (CX 23, Olympus, Japan).

## Results and discussion

### Characterization of Cet-SLN/ICG

**Figure 1.**
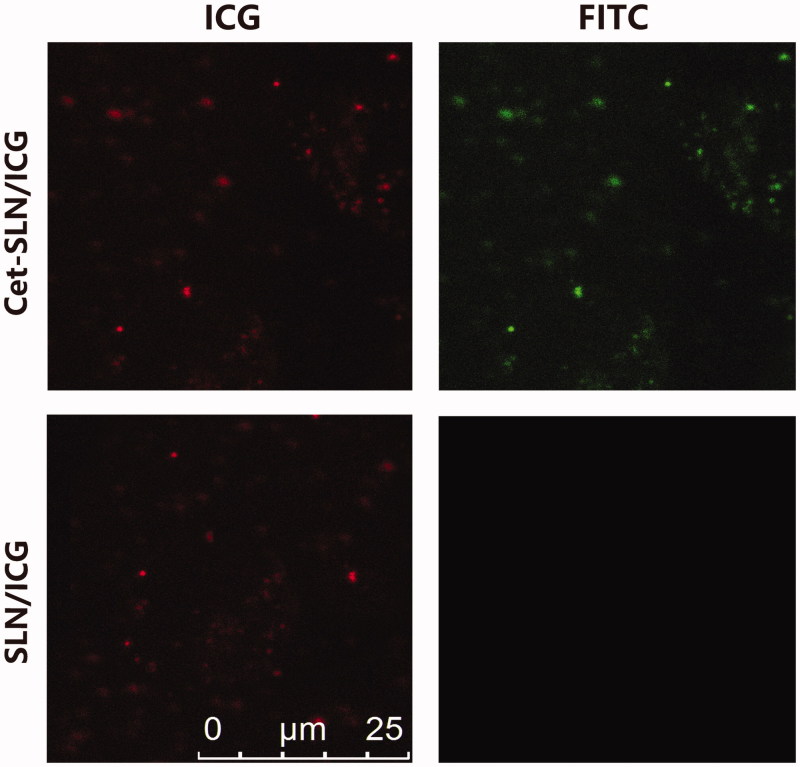
Successful conjugation of Cet to Cet-SLN. Scale bar: 25 μm.

The successful conjugation of Cet to SLN was firstly verified by fluorescent staining method. It was suggested that FITC-conjugated rabbit-anti-human IgG was able to bind with the surface conjugated Cet to induce aggregation of neighboring nanoparticles, which can be easily observed using fluorescence microscopy (Wang et al., 2017). As displayed in Figure 1, both green (from FITC) and red (from ICG) signals could be detected in the aggregates of Cet-SLN/ICG group which were highly merged. In contrast, only red signal can be observed in SLN/ICG group. These results indicated that Cet was successfully conjugated to the surface of Cet-SLN/ICG. In addition, the specific recognition also demonstrated that the surface conjugated Cet still preserved its bioactivity, which is prerequisite to exert its tumor-homing capability.

**Figure 2.**
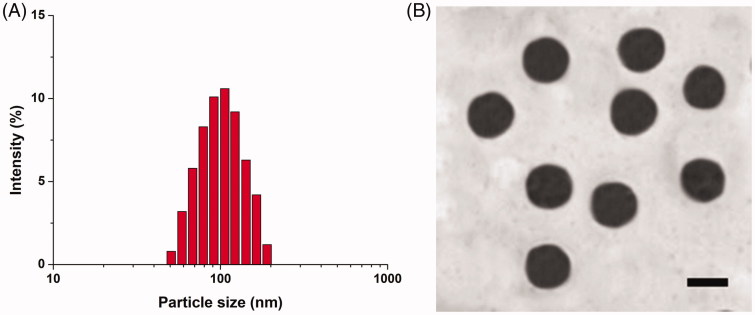
Particle size distribution (A) and TEM image (B) of Cet-SLN/ICG. Scale bar: 100 nm.

The particle size of Cet-SLN/ICG was shown in Figure 2(A). It revealed that Cet-SLN/ICG was nano-sized particle with homogeneous diameter at around 100 nm. Moreover, TEM image in Figure 2(B) revealed that Cet-SLN/ICG was spheroid nanoparticles with evident boundary, which further confirmed the conclusion obtained in Figure 2(A). In addition, it was noted that a change in zeta potential form positive (SLN/ICG) to neutral (Cet-SLN/ICG, data not shown) was also observed, further confirming the successful conjugation of Cet to the surface of SLN.

The conjugation of Cet on Cet-SLN was determined by UV spectrophotometer (14.52%). The DLC of ICG in Cet-SLN/ICG was 16.71% (data not shown).

### Stability and hemolysis assay

**Figure 3.**
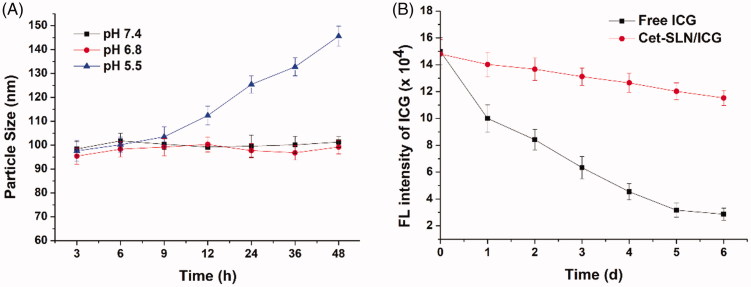
(A) Colloidal stability of Cet-SLN/ICG in PBS (pH 7.4, 6.8 and 5.5) at 37 °C for up to 48 h. (B) Comparative fluorescence stability of Cet-SLN/ICG and free ICG. Data were shown as mean ± S.D. (*n* = 3).

It has been well recognized that some basic requirements are to be satisfied if a DDS is intended to successfully deliver its cargos (You et al., 2018). Since the particle size plays critical role in determining the *in vivo* fate of the DDS, in order to bypass the complicated extracellular barriers, the DDS should be capable of maintaining its morphology (without significant size change) long enough before arriving at the targeted sites (Hashemi et al., 2016). As a result, Cet-SLN/ICG was studied regarding its stability as a function of time. To assess the colloidal stability of Cet-SLN/ICG under physiological conditions, the change in particle size in PBS with different pH was monitored for 48 h. As displayed in Figure 3(A), at pH 7.4 and 6.8, the size of Cet-SLN/ICG remained stable in all tested intervals with merely slight fluctuation. It was, therefore, suggested that Cet-SLN/ICG was able to maintain its size under physiological environment. In contrast, the stability of Cet-SLN/ICG was impaired under acidic conditions (pH 5.5), which might be beneficial for its drug release in tumor cells. On the other hand, it has been reported that ICG usually suffered from its irreversible rapid degradation in aqueous solution as well as risk of photobleaching. Preferable DDS should be able to protect the ICG from degradation in aqueous solution as well as under sunlight irritation. As a result, the fluorescence stability of Cet-SLN/ICG in compare to free ICG was studied for 6 days under sunlight. As shown in Figure 3(B), free ICG showed strong instability in aqueous solution under sunlight as its fluorescence intensity dropped significantly to 66% for merely 1 day after test and continuously declined until around 20% after 6 days. However, for Cet-SLN/ICG, the decrease in fluorescence intensity was dramatically retarded as only 22% of the ICG signal quenched after 6 days. These results clearly indicated that Cet-SLN/ICG could protect the encapsulated ICG from degradation as well as photobleaching. The preferable stability of Cet-SLN/ICG guaranteed favorable drug delivery, which is beneficial for its further development as a predictable and stable system to satisfy the requirements of other extensive applications in cancer therapy (Sheng et al., 2016).

### *In vitro* drug release

**Figure 4.**
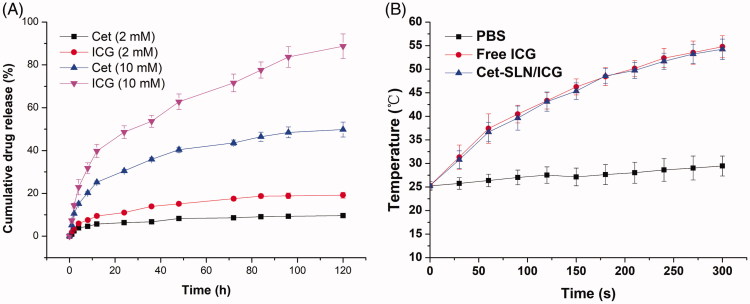
(A) Drug release profiles of Cet and ICG from the Cet-SLN/ICG in release media with extracellular and intracellular condition of GSH (2 mM and 10 mM). (B) Photothermal conversion capability of Cet-SLN/ICG. Data were shown as mean ± S.D. (*n* = 3).

Based on the following mechanisms, we expected that the as-prepared Cet-SLN/ICG might have a dual-responsive release behavior. Firstly, upon entering the cells, high intracellular GSH concentration might trigger ligand exchange to specifically release Cet. Later on, the detachment of Cet on the surface of the system might expose the encapsulated ICG that in turn facilitated its release. It was expected that Cet-SLN/ICG could remain stable in physiological environment (such as blood in the circulation system), while transfer to unstable to accelerate drug release within cancer cells. As a proof of concept, in our study, two different GSH concentrations (2 and 10 mM) were adopted to respectively mimicking extracellular and intracellular concentration of GSH. The release profiles of both drugs from Cet-SLN/ICG under different conditions were investigated and the results were summarized in Figure 4(A). As expected, it was observed that under extracellular environment (2 mM GSH), Cet-SLN/ICG was stable with both drugs being released less than 20% (120 h). On the contrary, significantly accelerated release was triggered under high GSH concentration (10 mM) for both Cet and ICG. It was interesting to note that the release of Cet and ICG showed a synchronous profile that slow release of Cet corresponded to slow release of ICG while faster release of Cet corresponded to a faster release of ICG. Since it was suggested that ICG was encapsulated within SLN while Cet was conjugated to the surface of SLN, we thereby supposed that the detachment of Cet could, in turn, facilitate the following release of ICG. This hypothesis deserves more exploration in our future works.

The photothermal conversion efficiency of Cet-SLN/ICG was evaluated by monitoring the temperature changes under laser irradiation. With the laser irradiation at 1 W/cm^2^ for 6 min, the temperature of Cet-SLN/ICG and free ICG significantly increased from room temperature (about 25 °C) to 54.78 and 54.23, while the temperature of PBS only increased 4.24 °C (Figure 4(B)), which indicated that Cet-SLN/ICG still reserve similar photothermal conversion capability to that of free ICG. It has been reported that temperature over 43 °C could exert irreversible damage to tumor cells (Saxena et al., 2004). As a result, it was expected that Cet-SLN/ICG could readily respond to the laser irritation and exert preferable anticancer effect *in vivo*.

### *In vitro* anticancer assay

**Figure 5.**
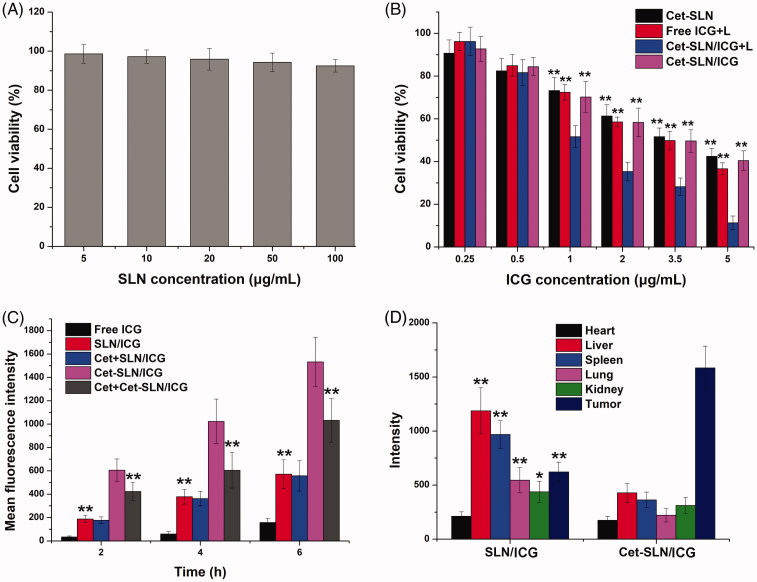
(A) Cytotoxicity of thiol-functionalized SLN after 48 h incubation with MCF-7 cells. (B) Cytotoxicity of Cet-SLN, free ICG (with laser irritation) and Cet-SLN/ICG (with or without laser irritation) against MCF-7 cells after 48 h incubation. **p < .01 versus Cet-SLN/ICG + L. (C) Quantitative analysis of intracellular time-dependent uptake of Cet-SLN/ICG against MCF-7 cells (pretreated with/without Cet). **p < .01 versus Cet-SLN/ICG. (D) Mean fluorescence intensity of dissected tumors and major organs of mice treated with Cet-SLN/ICG at 48 h post-injection. Data were expressed as mean ± S.D. (*n* = 3). *p < .05 and **p <.01 versus Cet-SLN/ICG.

The *in vitro* anticancer efficiency of Cet-SLN/ICG was investigated by standard MTT assay. It was suggested that cytotoxicity is a critical parameter to determine the safety profile of nanoparticles intended for *in vivo* administration. As a result, cytotoxicity of drug-free SLN was firstly assessed prior to anticancer assay. As shown in Figure 5(A), negligible cytotoxicity (more than 90% cell viability) was observed at the highest concentration of drug-free SLN, which suggested that the following cytotoxicity was not from carrier but the therapeutic agents. It was interesting to note that anticancer assay using drug-loaded nanoparticles in Figure 5(B) demonstrated that compared with Cet-SLN and free ICG (with laser irritation) groups, Cet-SLN/ICG with laser irritation showed the much more superior anticancer efficacy at all tested concentrations, while Cet-SLN/ICG without laser irritation simply achieved similar cytotoxicity to Cet-SLN. These results inferred that combined chemo-photothermal therapy of Cet and ICG hold superior cytotoxicity efficacy than Cet/ICG alone.

### Cellular uptake assay of Cet-SLN/ICG

Furthermore, we performed *in vitro* cellular uptake assay to study whether Cet modification could positively increase the nanoparticle internalization. Multiple previous researches have revealed that surface conjugation of Cet can improve the tumor-homing property of modified system to EGFR. As thereby suggested that MCF-7 overexpressing EGFR can be a preferable candidate to verify our conjecture (Wang et al., 2017; Tol et al., 2010; Xu et al., 2016).

As displayed in Figure 5(C), the fluorescence signal in cells increased as a function of time in both groups, suggesting a time-dependent cellular uptake of both nanoparticles. In addition, it was noted that both nanoparticles showed greatly increased intracellular ICG accumulation compared to free ICG, indicating that nanoparticles could significantly facilitate the delivery of ICG into cells. Moreover, it was observed that higher ICG signals were observed in Cet-SLN/ICG group at all tested time intervals, which was 2.68-fold of that in SLN/ICG group at 6 h post incubation. In order to verify whether internalization of Cet-SLN/ICG was via the EGFR mediated endocytosis, cells were pretreated with excess Cet for 2 h prior to addition of nanoparticles. It was interesting to observe that fluorescence intensity of Cet-SLN/ICG group suffered a great decline at all time intervals while that in SLN/ICG group remained almost the same level. It was clearly demonstrated by these results that Cet-SLN/ICG was internalized into cells through EGFR-related endocytosis.

### *In vivo* imaging assay

Cet modification was expected to help recognize the corresponding EGFR on cell surface and increase the homing of Cet-SLN/ICG to the tumor tissue. To verify our concept, the final distribution of ICG in mice bearing MCF-7 tumor was observed using a real-time imaging system at 48 h post single injection. Figure 5(D) showed the fluorescence distribution results obtained from *ex vivo* imaging of tumor and major organs. It was concluded that thiolated SLN was mainly distributed in the liver and kidney, which might due to their poor tumor targeting capability. On the contrary, Cet modification could significantly alleviate liver capture to assist the enhanced homing of ICG to tumor tissue.

### *In vivo* anticancer assay

**Figure 6.**
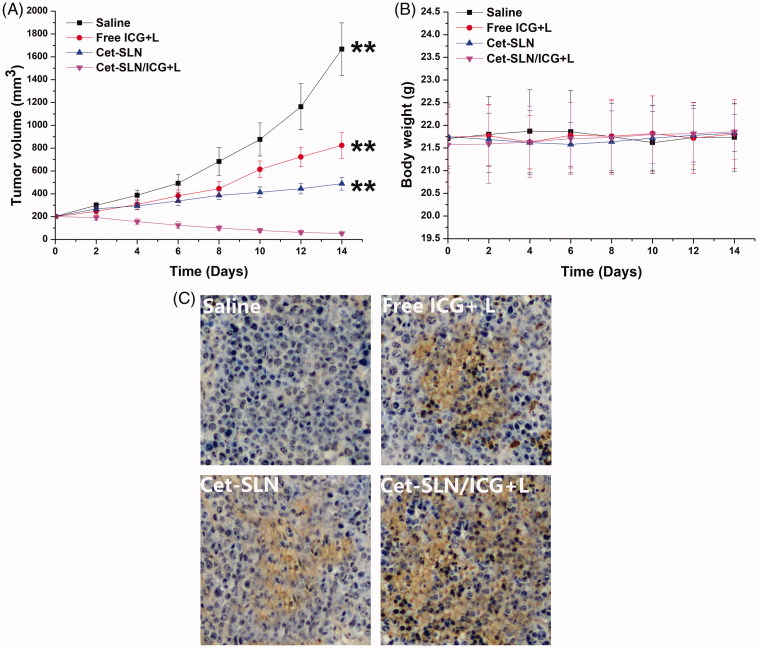
The tumor volume (A), body weight (B) and TUNEL staining of tumor tissue (C) analysis of MCF-7 tumor-bearing Balb/c nude mice after intravenous injection administration different formulations. Data were expressed as mean ± S.D. (*n* = 6). **p <.01 versus Cet-SLN/ICG.

*In vivo* anticancer assay of Cet-SLN/ICG was assessed. As shown in Figure 6(A), tumor growth was suppressed to a certain extent after treated with free ICG (with laser irritation) or Cet-SLN. However, the anticancer efficacy of mice in Cet-SLN/ICG (with laser irritation) group was much more effective than other counterparts and resulted in a greatly decreased tumor volume of approximately 52 ± 13 mm^3^. These results further demonstrated that synergetic effect of Cet and ICG was much more potent than monotherapy. Moreover, records of body weight variation of tested mice also revealed interesting phenomenon. As shown in Figure 6(B), as expected, there was no evident decline in body weight in mice treated with Cet-SLN/ICG, suggesting that tumor targetability of Cet-SLN/ICG could increase anticancer efficacy while reducing the side effects. In addition, Figure 6(C) indicated the TUNEL assays for tumor sections from different groups. Insignificant apoptosis was observed in saline group. In contrast, increased apoptosis was observed in all Cet and/or ICG containing groups. In addition, in line with results obtained in above assays, mice treated with Cet-SLN/ICG (with laser irritation) showed the most significant apoptosis with the best anticancer outcome as predicted. In summary, the Cet-SLN/ICG hold great promise as advanced tumor-targeting DDS for preferable breast cancer therapy.

## Conclusion

In our study, we successfully fabricated ICG loaded platform using Cet modified SLN (Cet-SLN/ICG). In this novel DDS, we combine the preferable tumor targetability of Cet as well as the preferable loading ability of SLN. Our results revealed that the as-prepared Cet-SLN/ICG was capable of specifically co-delivery Cet and ICG to achieve EGFR overexpressed MCF-7 cells. Moreover, the Cet-SLN/ICG was capable of releasing the loaded cargos in response to high GSH concentration. Moreover, both *in vitro* and *in vivo* experiments suggested that Cet-SLN/ICG hold superior tumor homing ability with much more effective anticancer efficacy than the mono systems due to the combinational chemo-photothermal effect.
